# Exploring the Relation Between Nursing Workload and Moral Distress, Burnout, and Turnover in Latvian Intensive Care Units: An Ecological Analysis of Parallel Data

**DOI:** 10.3390/ijerph22091442

**Published:** 2025-09-17

**Authors:** Olga Cerela-Boltunova, Inga Millere

**Affiliations:** Department of Nursing and Midwifery, Riga Stradiņš University, LV-1067 Riga, Latvia

**Keywords:** intensive care units, workload, burnout, professional, ethical dilemmas, personnel turnover, Latvia

## Abstract

Latvia faces one of the lowest nurse-to-population ratios in the EU, resulting in critical staff shortages in intensive care units (ICUs). Nurses frequently care for more patients than recommended, which not only compromises patient safety but also places heavy psycho-emotional burdens on staff. The aim of this study was to examine organizational-level relationships between objectively measured ICU nursing workload and subjectively reported psycho-emotional outcomes, including moral distress, burnout, and intention to leave one’s job. A secondary analysis combined data from two cross-sectional studies conducted in 2025. Workload was measured using 3420 Nursing Activities Score (NAS) protocols from three hospitals, while 155 ICU nurses from 16 units completed validated instruments assessing moral distress, burnout, and turnover intentions. The findings revealed persistent nurse shortages, with one ICU showing deficits exceeding 70% and mean NASs above 100 points per nurse per shift. Nurses reported moderate moral distress, particularly in situations of unsafe patient ratios and aggressive treatment, while burnout levels were moderate to high, especially in personal and work-related dimensions. About one-quarter of respondents were actively considering leaving their jobs. Moral distress significantly correlated with burnout (r = 0.357, *p* < 0.001), and organizational-level comparison indicated that higher workload was associated with greater emotional strain. These results not only highlight urgent national challenges but also resonate with international evidence on the link between unsafe staffing, moral distress, and workforce sustainability. Implementing systematic workload monitoring, safe staffing ratios, and structured support mechanisms is essential to safeguard ICU nurses’ well-being, reduce turnover, and protect patient safety in both Latvian and global contexts.

## 1. Introduction

The healthcare sector in Latvia is facing a significant shortage of professional staff, particularly nurses. According to international data, Latvia has one of the lowest rates of nurses per 100,000 population in the European Union, and this ratio continues to decline [1,2]. The critical situation is even more pronounced in inpatient care, where the workload per nurse often exceeds the acceptable norm, endangering both patient safety and the professional well-being of nurses [3]. Conditions are particularly challenging in intensive care units (ICUs), where nurses must provide care for patients with life-threatening conditions, using high technology, and often with limited resources [4].

Current global standards of care indicate that one nurse should not care for more than 1–2 patients at a time in the intensive care setting [5]. However, in practice in Latvia, a nurse often has to be responsible for 3–4 patients, especially at night or on weekends [6]. This level of workload not only affects the quality of care, but also causes significant physical and psycho-emotional fatigue [7]. International studies show that overload is closely linked to occupational burnout [8], moral distress [9] and intentions to change jobs [10]. In Latvia, there is not enough empirical data to allow for a well-founded analysis of this relationship and to link it to objective workload indicators.

Nursing workload is a complex, multidimensional construct that extends beyond patient numbers or shift hours. A concept analysis by Alghamdi (2016) identifies five defining attributes of workload: the amount of nursing time required, the level of professional competency, the weight of direct patient care, the degree of physical exertion, and the complexity of clinical activities [11]. Workload antecedents arise at the patient level (acuity, severity, and complexity of care), the nurse level (skills, experience, and coping capacity), and the organizational level (staffing resources, technology, and unit structure) [12]. The consequences of high workload are well documented, including elevated stress, moral distress, burnout, job dissatisfaction, absenteeism, and increased turnover, alongside reduced care quality and compromised patient safety [13]. In ICU, where patient acuity is consistently high and resources are frequently constrained, these dynamics are particularly pronounced, amplifying the risk of psycho-emotional strain and threatening workforce sustainability.

The work of ICU nurses is emotionally and ethically challenging, as they often have to make decisions in crisis situations, deal with dying patients, perform invasive manipulations, and face the desperation of family members [14]. In such circumstances, ethical dilemmas arise that nurses cannot always resolve due to systemic limitations, such as insufficient staff, time, or management support [15]. This results in moral distress—a state in which the healthcare worker knows what is right but is unable to act in accordance with their values [16]. Prolonged moral distress is associated with occupational burnout, emotional exhaustion, and loss of motivation [17].

These issues are being studied more and more actively at the international level. The COVID-19 pandemic further increased tensions in healthcare and highlighted the link between staff workload and burnout [18]. Studies in Canada, the USA, Sweden, and elsewhere indicate that up to 40% of intensive care nurses considered changing careers or leaving their jobs during the pandemic [19,20,21,22]. Moral distress has also been shown to often correlate with emotional exhaustion and burnout [23]. In response to this research, several countries have started to introduce mechanisms to measure workload and improve staff well-being [24].

One of the most widely used instruments to quantify the workload of ICU nurses is the Nursing Activities Score (NAS) [25]. This instrument allows for an objective assessment of how intensive nurses’ involvement in care has been during a specific period of time. Unlike the previously used TISS-28, the NAS includes not only technical manipulations, but also communication, documentation, and other care activities that are often underestimated. The total NAS allows determining what percentage of the work capacity of a nursing unit has been consumed, e.g., 75% NAS for one patient means that they require 75% of a nurse’s time during a 12 h shift [26].

In Latvia, the process of adapting the NAS instrument was initiated in 2024 [6], and one study analysed more than 3400 completed NAS protocols from hospitals of three different levels [27]. The results showed that the average workload exceeded 70%, and in more than half of the cases, more nurses were needed than were actually available. At the same time, significant staff shortages were identified, especially at night. These results are in line with international data, pointing to the need to measure workload regularly and plan resources on that basis [28].

In addition, a separate study on the prevalence of moral distress, occupational burnout, and intention to leave one’s job in Latvian ICU nurses was conducted with 155 respondents [29]. The study used validated instruments: MMD-HP (Measure of Moral Distress) [30], CBI (Copenhagen Burnout Inventory) [31] and ATS (Anticipated Turnover Scale) [32]. The results revealed a moderate level of moral distress, a high component of emotional exhaustion in burnout, and a significant number of nurses considering changing jobs in the next few years.

However, the two studies on workload [27] and psycho-emotional state [29] have been analysed separately so far. This study is the first attempt to pool the data and analyse how workload as measured by the NAS is related to nurses’ feelings, moral distress, and burnout. Although not collected in the same institutions, the data represent a common professional group over the same time period, with a similar care profile and working conditions.

To examine organizational-level relationships between objectively measured ICU nursing workload (NAS) and subjectively reported psycho-emotional outcomes (moral distress, burnout, and intention to leave) among ICU nurses in Latvia. The results obtained can serve as a basis for recommendations for improving staff management, implementing support mechanisms, and developing strategies for the well-being of ICU nurses in Latvia.

## 2. Materials and Methods

### 2.1. Study Design

This study was designed as a secondary analysis cross-sectional comparative study with the aim of analysing possible relations between the workload and psycho-emotional state of ICU nurses, such as moral distress, occupational burnout, and intention to leave one’s job. The study combines two previously conducted [27,29], mutually coordinated studies carried out as part of a doctoral thesis. One quantifying nurses’ workload using the NAS instrument [27] in three hospitals of different levels in Latvia, and another analysing the risks of moral distress, burnout, and staff turnover using structured and validated questionnaires [29]. Both studies were conducted in the same time period (February–May 2025), with identical ethical principles, rationale, and target audience of Latvian ICU nurses.

In this phase of the study, a secondary data analysis based on two previously published datasets is conducted to comparatively analyse workload data and psycho-emotional state indicators of ICU nurses, identifying possible thematic relationships between structural and emotional factors. Both datasets have been previously published in separate articles, but this analysis is the first attempt to put these results in a comparative and structurally linked context to identify parallels between workload and psycho-emotional risk factors.

### 2.2. Respondents

The study included two complementary participant groups: Workload data (NAS protocols) and survey data (ICU nurses).

Data on nursing workload were obtained from 3420 NAS protocols collected in three Latvian hospitals representing different levels of care (Hospital A: level III; Hospitals B and C: level II). Data were recorded in a structured electronic format during each 12 h shift. For each patient, care activities were documented using the validated Latvian version of the Nursing Activities Score (NAS) [25], where 100 points correspond to the workload of one nurse over a 12 h period. In addition to NAS values, information was collected on hospital type, shift type (day/night), number of nurses on duty, and the calculated staff shortage. All admitted patients during the observation period were included; no exclusion criteria were applied.

The second dataset included 155 ICU nurses from 16 ICUs across four Latvian regions (Rīga, Kurzeme, Vidzeme, Zemgale). Participants were recruited via ICU managers and professional associations. Inclusion criteria were: registered nurse with a valid professional certificate, active work in an ICU for at least three months, and direct patient care responsibilities. Nurses working exclusively in administrative or educational roles, or on long-term leave, were excluded.

To determine the minimum required sample size, an online calculator was used [33]. Assuming an estimated ICU nursing workforce of 500–600 nurses in Latvia, with a 95% confidence level and 10% margin of error, the recommended minimum sample was 96 participants. The final sample (n = 155) exceeded this requirement, ensuring sufficient power for correlational and regression analyses. Each participant completed three validated psychometric instruments adapted for Latvia:

- MMD-HP (Measure of Moral Distress for Healthcare Professionals): 27 items, two dimensions (frequency and intensity), 5-point Likert scales, total scores 0–216; higher scores indicate more frequent and intense moral distress. Cronbach’s α in this study: 0.938 (frequency), 0.976 (intensity) [30].
- Copenhagen Burnout Inventory (CBI): 19 items in three subscales (personal-, work-, and client-related burnout), 5-point Likert scale; higher scores indicate greater burnout. Cronbach’s α = 0.928 overall [31].
- Anticipated Turnover Scale (ATS): 12 items measuring intention to leave one’s job, 5-point Likert scale. Higher scores indicate stronger turnover intentions. In this study, Cronbach’s α = 0.342, suggesting multidimensionality; results are interpreted with caution [32].

Additionally, socio-demographic data were collected (age, gender, education, position, number of jobs, work schedule, and subjective workload perception) to identify risk factor profiles.

### 2.3. Staff Shortage Calculation

Staff shortage was calculated as the difference between the required number of nurses, based on NAS estimates, and the actual number of nurses present during each shift. According to the NAS methodology [6,25], 100 NAS points correspond to the workload of one nurse over a 12 h shift. For example, a shift with 1200 NAS points requires 12 nurses; if only 8 were present, the calculated shortage is 4 nurses.

In Latvia, there is currently no legally mandated ICU nurse-to-patient ratio. However, in clinical practice, a ratio of approximately 1 nurse per 2 patients is considered a safe minimum. This benchmark was used as a contextual reference when interpreting the calculated shortages.

### 2.4. Data Processing and Statistics

All data were analysed using IBM SPSS Statistics, version 28 (IBM Corp., Armonk, NY, USA). Descriptive statistics were calculated for all variables, including frequencies, means, standard deviations (SD), medians, minimum and maximum values, and 95% confidence intervals. Data normality was assessed using Shapiro–Wilk tests and visual inspection of histograms and Q–Q plots.

Group comparisons were performed using independent samples t-tests and one-way ANOVA for normally distributed variables. For non-normally distributed data, the Mann–Whitney U test and Kruskal–Wallis test were applied. Where significant ANOVA results were observed, Tukey’s HSD post hoc tests were conducted.

Associations between continuous variables were analysed using Pearson’s correlation coefficient (r) for normally distributed data and Spearman’s rho (ρ) otherwise. The interpretation of correlation strength followed Cohen’s conventional thresholds: small (0.10–0.29), medium (0.30–0.49), and large (≥0.50).

To identify predictors of moral distress, burnout, and turnover intention, multiple linear regression analyses were performed with socio-demographic and professional factors as independent variables. In addition, a simple mediation model (PROCESS Macro v4.1, Model 4) [34] was used to test whether burnout mediated the relationship between moral distress and turnover intentions, applying 5000 bootstrap samples with 95% confidence intervals.

An organizational-level aggregated analysis was applied, combining unit-level workload data (NAS) with survey-based psycho-emotional outcomes. As the datasets are independent, inferences are made at the work-environment level, not at the individual level. This design combines two independent datasets: workload data (NAS protocols) and survey-based data (moral distress, burnout, turnover intentions). As these datasets are not linked at the individual nurse–patient level, analyses were performed at an aggregated level (e.g., average workload in hospitals compared with average psycho-emotional indicators of ICU nurses). This approach allows for contextual interpretation of systemic associations between staffing levels and nurses’ well-being but does not permit causal or individual-level inferences.

All statistical tests were two-tailed, with a significance level of *p* < 0.05.

### 2.5. Ethical Considerations

Both studies were carried out with the approval of the Ethics Committee of Riga Stradiņš University (decision No. 2-PĒK-4/416/2023, 9 May 2023). All data were collected ensuring complete anonymity of participants, voluntary participation, and informed consent. Data processing was carried out electronically, adhering to all principles of personal data protection [35].

## 3. Results

### 3.1. Nursing Workload in ICUs

The workload assessment was performed by analysing 3420 NAS protocols from three Latvian medical treatment institutions, Hospital A, B and C, representing different levels of ICU care. The NAS data provide an objective picture of the volume of care activities per nurse during a 12 h shift. The results of this analysis are summarised in Table 1, which includes NAS means, standard deviations, workload thresholds, current staffing levels, as well as the estimated nurse shortage in both absolute and relative terms.

Hospital A had relatively similar workload values for both day and night shifts: 58.38 (SD = 20.14) and 58.13 (SD = 19.20) NAS points per nurse on average, respectively. Each shift was covered by 8 nurses, but based on NAS data, it has been calculated that an additional 0.97 nurses per day shift and 0.95 nurses per night shift are optimally required, representing a deficit of 32.4% and 31.7%, respectively, compared to the required number.

Hospital B had the highest workload level among all the units compared. The mean NAS was 106.96 (SD = 18.83) during the day shift and 105.18 (SD = 18.99) during the night shift. Only 2 nurses covered each shift, but according to the NAS norms, an additional 2.46 nurses during the day shift and 2.19 nurses during the night shift would have been required. This represents a critical staff shortage of 82.1% during the day shift and 73.0% during the night shift, which significantly exceeds the limits.

The workload at Hospital C was average compared to other units, with 71.31 (SD = 24.61) during the day shift and 70.85 (SD = 19.69) during the night shift. There were 2 nurses on shift, but it was estimated that an additional 1.32 nurses would be needed during the day shift and 1.14 nurses during the night shift, corresponding to a nursing shortage of 44.0% and 38.0%.

A nursing staff shortage was identified in all three hospitals, most notably in Hospital B, where each nurse has to take on a significantly excessive workload. The workload was lower at Hospital A, but the shortage was still more than a third of the required staff. These data illustrate the mismatch between actual staffing and clinical care needs, which potentially affects the quality of care and nurses’ psycho-emotional well-being.

### 3.2. Psycho-Emotional Stress and Staff Turnover Intentions

A total of 155 ICU nurses from Latvian hospitals participated in the psycho-emotional assessment, completing three validated instruments: MMD-HP [30], CBI [31], and ATS [32]. The sample comprised 155 ICU nurses; 96.1% female; age 21–63 years; mean professional experience 17.9 years.

#### 3.2.1. Moral Distress Results

To assess the prevalence and intensity of moral distress among Latvian ICU nurses, the 27-item MMD-HP [30] version was used. The measure consists of two separate dimensions, frequency of occurrence and intensity of emotional impact, which allow to assess both how often nurses encounter potentially ethically difficult situations and how emotionally impactful these situations are.

The means in the section on how often nurses encounter moral distress issues ranged from 0.57 to 2.35, revealing that certain situations are experienced significantly more often by nurses than others. The most frequently noted situations were as follows:

1. Question No. 5: continuation of aggressive treatment when the patient’s death is highly probable (M = 2.35);
2. Question No. 16: obligation to care for more patients than is safely possible (M = 2.18);
3. Question No. 19: excessive documentation requirements that compromise the quality of care (M = 2.26).

These situations represent clinically and ethically complex scenarios in which nurses experience professional and emotional stress, possibly coming into conflict with their own values. Overall, the results of the measure reveal that Latvian ICU nurses regularly face ethically challenging situations, which the measure is able to identify with statistical reliability.

The section of the measure on the intensity of moral distress showed a pronounced psychological impact on the respondents. The overall mean result of the measure was M = 94.30 (SD = 40.03), with a value range from 0 to 97, indicating a large difference in the emotional response to different situations among respondents, with some nurses feeling very little impact, while others experience high psychological stress. The mean values of the individual items in the emotional intensity section ranged from 1.54 to 2.63. The situations with the most intensive emotional impact were as follows:

1. Question No. 16: insufficient staff—too many patients per nurse (M = 2.63);
2. Question No. 8: participation in care that causes unnecessary suffering (M = 2.52);
3. Questions No. 5, 9, 14: related to aggressive treatment, lack of continuity of care, and poor team communication conditions (all M > 2.4).

These situations shed light on organisationally and ethically problematic realities of care, where the professional values and emotional resilience of nurses are put under pressure.

Summarising the results, it can be concluded that Latvian ICU nurses regularly face professionally challenging situations that cause emotional stress and moral conflict. Both the frequency and intensity dimensions showed structurally reliable and clinically relevant findings.

#### 3.2.2. Copenhagen Burnout Inventory Results

To assess the phenomenon of burnout among ICU nurses, the CBI [31] was used, which includes three subscales: personal-related burnout (PRB), work-related burnout (WRB), and client-related burnout (CRB).

The total CBI [31] score was 37.59 out of a possible 76, or approximately 49.5% of the maximum potential, indicating a moderate level of burnout among Latvian ICU nurses. This indicator is dangerously close to the 50% threshold, which is often considered in international literature [30] the threshold beyond which burnout begins to manifest itself as real clinical and professional symptoms, such as demotivation, mistakes at work, or the intention to change or leave the job [10,13]. The scores obtained ranged from 10 to 64 points (SD = 11.945, SE = 0.959), confirming a high diversity of individual experiences.

In the PRB subscale, the average score was 2.35, which corresponds to 58.75% of the maximum possible level. This indicator reflects a moderately high level of personal burnout and indicates frequent fatigue, physical and emotional exhaustion, and a decline in professional motivation.

In the WRB subscale, the average score was 2.20 or 55% of the maximum burnout potential. The results can be interpreted as moderate burnout, mainly related to the work environment, prolonged overload, lack of management support, or ineffective work organisation. A large range of standard deviations indicates high individual variability, which points to unequal experiences of nurses across hospitals and work shifts.

In the CRB subscale, the average score was 1.63, or 40.75%, the lowest of the three dimensions. These results indicate that the level of emotional exhaustion towards patients is relatively low and that most nurses are able to maintain empathy and professional distance even in difficult situations.

In all three CBI [31] dimensions, burnout indicators exceed 40%, while PRB and WRB exceed 50%, which is considered a sign of systematic psychological overload. These indicators are clinically significant and confirm the need to introduce targeted preventive measures at both organisational and professional support levels.

#### 3.2.3. Potential Staff Turnover

In this phase of the study, a 12-question scale [32] was used to assess ICU nurses’ potential intention to change jobs. The overall scale mean was M = 42.60 (SD = 8.378; SE = 0.672), with a range of scores from 14 to 64, indicating significant variability among respondents in their intention to stay or leave their current job. This dispersion reflects both a high sense of professional belonging for some nurses and a strong desire to change jobs for others.

The mean values for individual questions ranged from 1.80 (Question No. 12: I plan to leave this job soon) to 4.67 (Question No. 7: I have been in this job for as long as I wanted to be), which indicates the highest level of agreement with statements about belonging and satisfaction. However, a number of questions directly related to the willingness to leave one’s job had lower scores, indicating that some respondents are considering a job change or have already made a decision to do so.

#### 3.2.4. Results of Correlation and Regression Analysis

To understand the psycho-emotional state of intensive care nurses and the factors influencing it, the interrelationships between the three instruments and the predictors of these constructs were analysed using correlation, linear, and logistic regression, and analysis of variance.

Pearson correlation analysis demonstrated a moderately strong positive relationship between moral distress and burnout (r = 0.357, *p* < 0.001), confirming the theoretical expectation that greater moral distress is associated with higher levels of burnout across all its dimensions.

Potential staff turnover, on the other hand, did not significantly correlate with either burnout or moral distress (r < 0.10; *p* > 0.05), suggesting that other environmental and organisational factors also influence the decision to leave one’s job. The absence of significant correlations between turnover intentions and other psycho-emotional indicators may be explained by methodological limitations, including the relatively small sample size, the use of heterogeneous instruments, and the influence of contextual organisational factors not captured in the present study.

Three linear regression models were constructed, each using one of three indicators (MMD [30], CBI [31], ATS [32]) as the dependent variable. All models were statistically significant and showed a different set of impact variables. Logistic regression showed that moral distress significantly reduced the willingness to take on additional responsibilities (β = −0.038; *p* < 0.001). Each point increase in moral distress reduces the probability of this by about 3.7%.

### 3.3. Organizational-Level Comparison Between Structural Stress and Psycho-Emotional State

To analyse whether structural workload indicators in ICUs correlate with indicators of nurses’ psycho-emotional well-being, an Organizational-level comparison was performed by combining data from ICUs of three Latvian hospitals. NAS data were obtained from shift registers, while MMD-HP [30], CBI [31] and ATS [32] were obtained through an anonymous cross-sectional survey.

Preliminary analysis indicates that ICUs with high total NAS [25] scores (above 100) and staff shortages above 70%, such as Hospital B, also had higher mean values in the psycho-emotional distress indicators, especially in the work-related burnout and moral distress subscales. In contrast, Hospital A, where staff coverage was better and NASs were lower (~58 points), showed comparatively lower levels of burnout and distress.

**Figure 1:**
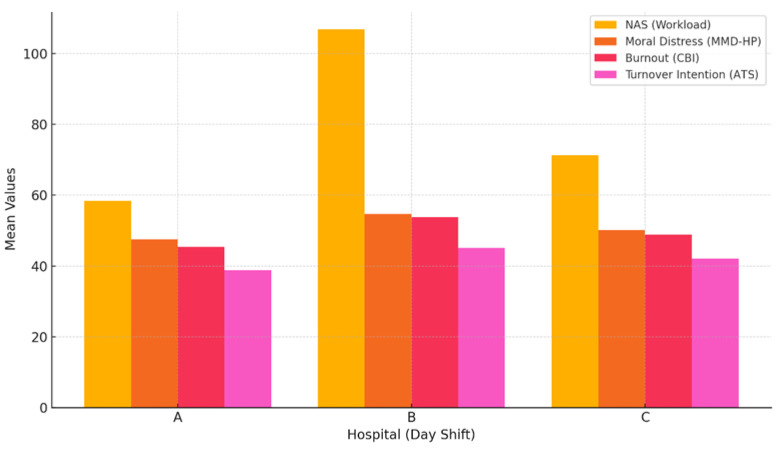
Comparison of NAS Workload and Psycho-Emotional Indicators (organizational-level). Abbreviations: NAS—Nursing Activities Score; MMD-HP—Measure of Moral Distress for Healthcare Professionals; CBI—Copenhagen Burnout Inventory; ATS—Anticipated Turnover Scale.

Although the number of units is limited (n = 3), the visual trend suggests that there is a conceptual relationship between higher structural load and higher psycho-emotional risk (Figure 1). Spearman’s correlation analysis between NAS [25] and moral distress indicators at the unit level showed moderately strong but statistically insignificant correlations (ρ = 0.60; *p* > 0.05), which can be explained by the small number of units and methodological limitations (Table 2).

In Hospital B, where the highest mean NAS workload (D:M = 106.96; N:M = 105.18) and also the highest nurse shortage (D: 82.1%; N: 73.0%) were recorded, the highest mean values were also found for moral distress (54.7), burnout (53.8) and potential staff turnover (45.1). This indicates a close link between high workload, staff shortage, and risks to psycho-emotional well-being.

In contrast, in Hospital A, where the NAS mean values were lower (D: 58.38; N: 58.13) and staff shortage was significantly lower (32.4–31.7%), lower rates of moral distress (47.6), burnout (45.4), and staff turnover (38.9) were also observed, suggesting a possible protective effect of better work organisation.

Hospital C occupied an intermediate position with an average NAS workload (D: 71.31; N: 70.85) and a moderate staff shortage (44.0–38.0%), which was also reflected in average moral distress (50.1), burnout (48.9), and turnover (42.1) indicators.

This organizational-level comparison illustrates the structural differences between ICUs and their potential impact on nurses’ psycho-emotional well-being, highlighting the need to address staff shortage and overload as key factors associated with moral distress and occupational burnout.

Figure 1 illustrates the relationship between NAS and mean values of moral distress, burnout, and staff turnover in hospitals. There is a trend towards higher workload being associated with higher psycho-emotional risks.

**Figure 2:**
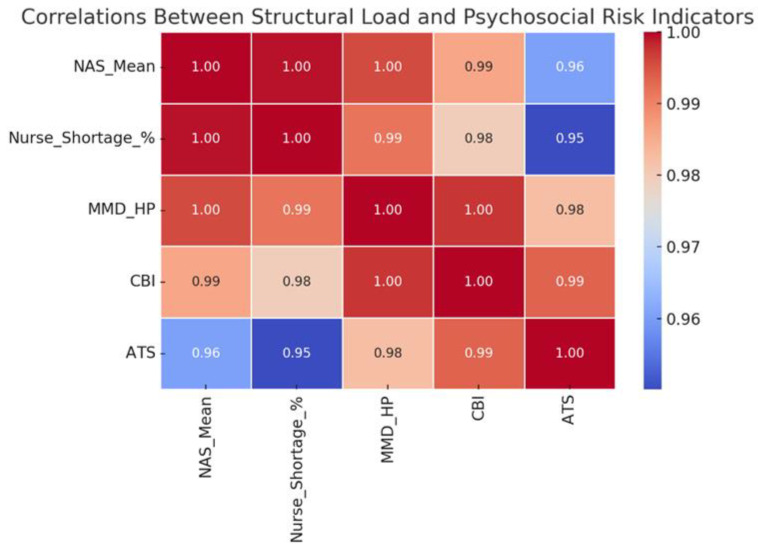
Correlations Between Structural Load and Psycho-emotional Risks in ICUs (organizational-level). Abbreviations: NAS—Nursing Activities Score; MMD-HP—Measure of Moral Distress for Healthcare Professionals; CBI—Copenhagen Burnout Inventory; ATS—Anticipated Turnover Scale.

The correlation map in Figure 2 shows the relationship between the structural load indicators (mean NAS and the three main psycho-emotional indicators). Although the number of units analysed is small, the visualisation shows conceptual trends, with higher structural load tending to be associated with increased psycho-emotional risk. This approach allows the identification of systemic factors that can affect the emotional well-being of staff.

Although n = 3 limits generalisability, these data serve as a pilot analysis for further research where a larger sample of units and data collection over more time units is needed.

## 4. Discussion

The results of this study confirm that psycho-emotional risks for ICU nurses are closely related to working conditions and workload. High workload, characterised by high NASs, has a negative impact on nurses [36]. Overload contributes to burnout, namely emotional exhaustion and job dissatisfaction, which in turn increases the desire to leave the job [6]. The literature consistently notes that overload and inadequate support at work lead to chronic stress and accelerate staff turnover [37]. In our study, more than half (53.5%) of ICU nurses have considered leaving because of moral distress and a quarter (25.2%) were actively considering it. These figures are worrying, but they are in line with international trends. For example, an ICU staff survey in Spain found that employees with higher levels of moral distress were more likely to have an intention to leave their job [38]. Available data also suggest that around one in three ICU nurses in Europe plan to leave within the next few years, with rates approaching 50% in some countries such as Poland and Spain [38,39]. This shows that the problem of staff retention in ICUs is global and linked to work environment factors.

The data obtained in the study confirm a significant positive correlation between nurses’ moral distress and occupational burnout (r = 0.357, *p* < 0.001). This means that the more moral distress a nurse experiences, the higher the burnout indicators. This finding is consistent with studies conducted in other countries [40,41]. For example, in a Brazilian sample of university hospital nurses, the intensity and frequency of moral distress was significantly associated with components of burnout syndrome, particularly high emotional exhaustion [40], and nurses with a low sense of professional achievement and high exhaustion had higher levels of moral distress. Systematic reviews have also concluded that moral distress is a serious problem with a high prevalence, negatively affecting the health of both staff and patients [13,20,41]. The main causes of moral distress are situations where nurses are unable to act in accordance with their professional and ethical beliefs due to institutional constraints [41]. Common factors include inappropriate management policies, excessive working hours and staff shortage, as well as incompetence of colleagues or lack of support. All of these are powerful sources of moral suffering. The nurses surveyed in this study regularly faced ethically challenging situations, and more than half admitted to experiencing moral discomfort that made them question their future in the profession. The chronic effects of such moral distress contribute to emotional exhaustion, the development of cynicism, and low job satisfaction, resulting in an increase in burnout syndrome [40,41]. The consequences of burnout manifest themselves not only in a deterioration of well-being, but also in professional performance. Studies show that burnt-out nurses make more mistakes and are less compassionate towards patients, which compromises the quality of care and patient safety [42].

The findings of the study are generally comparable to studies conducted in Europe and other regions, although there are some contextual differences. The prevalence of professional burnout among ICU nurses in Europe is very high. For example, a study conducted in Belgium in 2020 showed that 68% of ICU nurses were at risk of burnout during the pandemic [43]. In particular, 38% experienced severe emotional exhaustion and about 30% experienced high depersonalisation, while subjectively increased workload and unfavourable working conditions such as poor staff/patient ratio (1:3) significantly increased the risk of all dimensions of burnout [43]. These indicators are consistent with observations from our study, where staff shortage and high workloads were associated with higher burnout and distress. Similar trends have been reported elsewhere in Europe. A 2025 survey of five countries (Poland, Spain, Croatia, Romania, and Cyprus) found that nearly one-third of ICU nurses were considering leaving their job in the near future, with nearly half in Spain and Poland [39]. These data show that the Latvian results, with 25% of nurses currently considering leaving, are in the same range of the problem. Similarly, Spanish researchers have found that ICU staff with a poorer ethical climate and higher levels of moral distress have a significantly higher willingness to leave their jobs [38]. In our study, morally distressed nurses considered leaving more than twice as often, which supports the Spanish finding that employees with high levels of distress feel compelled to look for another job [38]. Internationally, this is consistent with meta-analysis data estimating that, on average, ~28% of intensive care nurses worldwide plan to leave their jobs [44]. It should be noted that the COVID-19 pandemic exacerbated the situation in many countries. Studies have described how moral distress increased during the pandemic and how many additional factors, such as fear of infection and lack of resources, contributed to staff burnout and attrition [40]. However, similar patterns were also observed before the pandemic. The intensive care environment with seriously ill patients, frequent ethical dilemmas, and a high level of responsibility is in itself a high risk factor for nurses’ well-being [41]. Thus, the psycho-emotional well-being indicators of Latvian ICU nurses and their relationship to the work environment is in line with the situation in Europe, where staff overload, moral conflicts, and burnout are widely documented problems [6,45].

The findings of this study and other authors allow us to identify a number of key factors that critically influence the psycho-emotional well-being of ICU nurses and their willingness to leave their jobs. First, workload and staff shortage stand out as primary sources of stress [46]. If one nurse has to care for too many seriously ill patients, safe capacity is exceeded, as shown, for example, by a study conducted in France and Belgium where 71.4% of nurses had shift loads exceeding 100% NAS, indicating a chronic shortage of human resources and potentially compromising patient safety [43,47]. Our study also found that higher intensity of patient care is often associated with fewer available staff. A moderately strong negative correlation was found between NAS episode scores and the actual number of nurses on shift (r = –0.49; *p* < 0.001), i.e., staff shortage was more pronounced in the most intense situations. This result indicates that staff shortage at times of peak workload can cause the greatest harm to nurses’ well-being.

Among psycho-emotional factors, moral distress and professional burnout have the greatest impact. Our data show that nurses have a significantly higher desire to change jobs, and in regression analysis, one of the strongest predictors of intention to leave was previous moral discomfort at work. This means that if a nurse has previously felt compelled to consider leaving, for example, due to ethical conflicts or exhaustion, this experience greatly increases the risk of actually leaving in the future.

Occupational burnout also contributes directly to staff turnover. Studies show that with each unit increase in emotional exhaustion, the likelihood that a nurse will leave the job in the near future increases by 12% [48]. The CBI data we obtained correlate with these findings that respondents with higher burnout scores were also more likely to have thoughts of leaving the profession. Moreover, burnout and moral distress are mutually reinforcing, creating a vicious circle. Burnt-out nurses feel more morally vulnerable, while prolonged moral suffering contributes to cynicism and depersonalisation [41], thus accelerating the burnout process.

In addition to workload and ethical stressors, support from management and colleagues, the ethical climate in the workplace and opportunities for professional development are also important influencing factors. For example, a Spanish ICU study found that a better ethical climate in the hospital was inversely related to moral distress (r = −0.277), so a supportive, open team environment reduces the intensity of moral distress [38]. Our results similarly indicate that nurses who felt the need for preventive support measures and who did not receive sufficient supervision were more likely to experience moral distress and a desire to change jobs.

Among socio-demographic factors, education and marital status played a statistically significant role in this study, but their impact on levels of distress and burnout was smaller than that of work environment factors. Overall, the main elements that threaten the psycho-emotional well-being of nurses are those that interfere with the quality of their professional duties, such as overload, unresolved ethical conflicts, lack of emotional support, and chronic fatigue. These same factors are also the strongest drivers of nurses’ desire to leave the job or even the profession [6,45].

The study also provided insights into the differences between intensive care units (hospitals) by comparing NASs, staff shortage, and psycho-emotional indicators across units. The analysis revealed that there are systematic differences between hospitals, indicating the role of organisational factors. For example, in one of the hospitals included in the study (Hospital A), the median shortage of nurses was around 9 nurses per shift, which is significantly higher than that observed in other units. This means that Hospital A was short of almost ten nurses during a typical shift to provide optimal care, based on the patient severity profile (NAS). In other words, staffing levels there consistently lagged behind actual needs. In contrast, in another hospital (C), the median shortage was close to zero, indicating a relatively balanced allocation of resources. Interestingly, at Hospital A, despite the highest staff shortage, the average NAS workload was the lowest and the most consistent of all. This may suggest that Hospital A may be operating with fewer intensive care beds or lower patient complexity, but due to staff shortages, even moderate workloads are chronically understaffed. Hospital B, on the other hand, had the consistently highest NAS workload and nurses were regularly required to care for very seriously ill patients, but the median staff shortage was lower in Hospital B (2–3 nurses). It is possible that Hospital B formally has more staff positions or more effective staff planning, which partially compensates for the heavy workload.

These ecological observations indicate that organisational policies and resource planning have a significant impact on nurses’ working conditions. In units with chronic understaffing, nurses are likely to accumulate both physical and moral fatigue, which may manifest itself in higher levels of burnout and distress. Although psycho-emotional indicators were analysed mainly on an individual basis in our study, comparative data suggest that nurses at Hospital A, with the greatest staff shortage, might have higher moral distress and lower satisfaction compared to Hospital C, where staffing adequacy was much better. International examples support this interpretation. A comparative study of several hospitals in Belgium [43,49] concluded that the standard intensive care staff/patient ratio of 1:3 is not sufficient to provide quality care and, according to NAS calculations, an average ratio of 1:1.5 (one nurse per 1–2 patients) would be necessary. This is consistent with our estimate that in units with high NAS workload, significantly more nurses than formally required should be provided for optimal care. Ecological analysis thus highlights systemic deficiencies: if nurses in one hospital consistently have to care for more patients than is physically possible, staff in that unit are exposed to more emotional distress and burnout. The practical implication of this data is that healthcare managers need to regularly compare workloads and staffing indicators between departments to identify problem areas and prevent critical overload. Our study shows that data on NAS and staff shortage can serve as a reliable ‘early warning’ signalling the need to intervene before high staff workloads lead to massive staff attrition or patient safety risks.

Based on the findings of the study, a number of recommendations can be formulated to improve the psycho-emotional well-being of ICU nurses and reduce staff turnover. Firstly, personnel planning based on actual workload is necessary. Systematic monitoring of nursing workload using validated NAS instruments or similar should be introduced. Our results clearly showed that objective measurement of workload allows identifying times and places where nurses are overloaded [6]. International experience shows that the introduction of NAS helps to more accurately determine the number of nurses needed according to patient needs, which reduces burnout and improves the quality of care [6,43]. Therefore, policies should provide national guidelines for optimal staffing levels in ICUs based on workload data. For example, if the NAS regularly exceeds 100% per nurse, additional staff positions must be provided. The study found that the introduction of NAS in Latvia can serve as a basis for data-driven decision-making, optimising the allocation of nurses and avoiding situations where overload is combined with lack of support [6].

Secondly, a safe norming of nurse-to-patient ratios should be introduced. Policy makers should consider introducing stricter regulations on the maximum number of patients per ICU nurse. As mentioned, studies in Europe show that a 1:3 ratio is often detrimental to both staff and patients [6,25,34,43,47]. In complex patient care, the optimal ratio should be close to 1:1 or 1:2. Reviewing the regulations and adapting them to the actual workload, taking into account the measurements of NAS or similar instruments, would be a strategic step towards reducing the unbearable burden placed on nurses. This would directly address one of the main causes of burnout—overload—and thus also improve staff retention in the long term [6].

Thirdly, healthcare institutions need to put in place systematic support measures to reduce the effects of moral distress and burnout. Our study revealed that the need for preventive measures was one of the themes that emerged from the surveys. This can include regular supervision and debriefing sessions for ICU staff after serious ethical cases, where nurses can discuss emotionally difficult situations in a safe environment. Evidence also shows that strengthening the moral resilience of nurses can yield positive results. Studies following COVID-19 have shown that higher moral resilience is associated with lower rates of burnout and even ‘quiet quitting’ [45]. Institutions can introduce training and support groups to deal with moral distress, developing nurses’ capacity to cope with ethical conflicts. It is also essential to have day-to-day management support and to create an environment where nurses feel heard and involved in decision-making, especially on issues that affect ethical dilemmas and the quality of patient care. A study in Spain recommended improving the ethical climate in ICUs specifically, as this can significantly reduce the level of moral distress and, consequently, the desire to leave the job [38]. Similarly, we recommend that hospital managers strengthen team cohesion and open communication, as this helps nurses feel psychologically safer and reduces work-related tensions.

Fourthly, measures should be put in place for the early diagnosis and prevention of occupational burnout. Employees at high risk of burnout can be offered more flexible schedules, additional leave, or rotation to less intensive units temporarily to prevent total exhaustion [50]. As burnout was significantly associated with intentions to leave one’s job in our study, early recognition of burnout syndrome is critical for staff retention. It is also internationally recommended to increase remuneration and social benefits specifically for ICU staff, recognising the increased workload and emotional costs of these jobs [51]. Adequate pay and career growth opportunities can reduce a nurse’s desire to leave the job, even if the day-to-day is difficult, by increasing the sense of professional satisfaction.

In summary, the study highlights a critical need for systemic improvements in the working conditions of ICU nurses. Overload, moral distress, and burnout are interlinked and together contribute to staff turnover, solutions must be comprehensive and aimed at reducing these factors. At policy level, this means investing in human resources, developing evidence-based standards on workload and staffing levels, and introducing support mechanisms that address the mental health of staff. The findings of our study are in line with international recommendations that, for example, accurate measurement of workload and monitoring of staff well-being can serve as a basis for early intervention, preventing both nurse burnout and patient safety risks [6]. In the long term, by improving the working environment and providing the support that nurses need, the healthcare system will not only have a more resilient and motivated staff, but also a higher quality of patient care [6].

This study has several important implications for nursing practice, healthcare management, and policy development. First, the findings demonstrate that nurse staffing shortages in Latvian ICUs are not only persistent but frequently exceed internationally recommended safety levels. This highlights the urgent need to introduce systematic workload monitoring, for example, through regular use of the NAS, to provide objective evidence for workforce planning and allocation.

Second, the relation between moral distress, burnout, and turnover intentions underscores the need for comprehensive support mechanisms at both organizational and national levels. Interventions such as structured debriefing sessions, ethical consultation services, and programs to strengthen resilience could mitigate the psycho-emotional burden on nurses [52,53].

Third, the study provides an evidence base for developing safe staffing policies in Latvia, where no legally mandated ICU nurse-to-patient ratio currently exists. Establishing clear standards, such as a minimum 1:2 nurse-to-patient ratio, would align Latvian practice with international recommendations and improve both patient safety and staff well-being [44].

Finally, by combining objective workload data with subjective assessments of moral distress and burnout, this study offers a model for integrated workforce research. The organizational-level comparison approach illustrates how different types of data can be meaningfully combined, even when collected independently. This methodological contribution may be useful for future studies in other countries where workload monitoring systems are still developing.

Although we initially used the term “partially ecological”, the design is best understood as an organizational/work-environment analysis: we compared aggregated unit-level NAS data with survey outcomes. No population-level ecological inference is intended. The evaluated variables—including workload, moral distress, burnout, and turnover intentions—are all related to the work environment of ICU nurses. The ecological aspect does not imply population-level epidemiological inference but rather reflects the aggregated comparison of institutional workload data with survey-based psycho-emotional outcomes. Therefore, the study should be interpreted primarily as an organizational analysis of nursing conditions in Latvian ICUs, providing insight into systemic associations between staffing shortages and nurses’ well-being.

## 5. Limitations

This study, which combines data from two separate studies, has several important limitations. First, data on workload and emotional indicators have been collected over different time periods and in different hospitals in Latvia. The NAS data cover three specific hospitals, while the moral distress and burnout data come from nurses in 16 ICUs, which do not necessarily overlap. It is therefore not possible to make a precise individual comparison for each nurse or unit, but only to analyse possible general directions of correlation.

Second, the two datasets are based on different types of measuring instruments. The NAS is an objective workload measurement scale that is completed according to the patient care protocol, while moral distress, burnout, and intention to leave the job were assessed by self-assessment questionnaires based on subjective experience. Such data heterogeneity can affect the interpretation of correlations and complicate the identification of precise causal relationships. The internal consistency for the ATS in this study was low (Cronbach’s α = 0.342), suggesting multidimensionality and warranting cautious interpretation of intention-to-leave findings.

Third, the correlation design does not allow conclusions to be drawn about cause and effect. This study can establish relationships between variables, but not prove that workload causes moral distress or burnout. Potentially influencing confounding factors, such as management support, team microclimate, or individual resilience, were not included in the analysis.

Fourth, the data were collected over specific time periods and may not reflect long-term trends or seasonal workload variations. For example, the NAS data were collected over a three-month period, which may not reflect the overall workload dynamics over the year.

Fifth, survey data may be at risk of social-desirability bias. Nurses may have underestimated or overly positively estimated their emotional state for fear of identification or stigmatisation. Although anonymity was guaranteed, subjective surveys always carry this risk.

Despite these limitations, the study provides valuable initial insights into the potential links between workload and emotional risks in the context of Latvian ICUs. This serves as a basis for further studies with a more integrated design, a longer data collection period, and additional control of variables.

## 6. Conclusions

This study provides important insights into the link between structural workload and psycho-emotional well-being among Latvian ICUs. Combining data from objective workload indicators (NAS) and subjective experience measures (moral distress, occupational burnout, and intention to change jobs), it was found that overload and insufficient staffing correlate significantly with higher emotional risks. Higher NAS workload and higher staff shortage were associated with higher rates of moral distress and burnout, while moral distress and burnout were mutually reinforcing and had a significant impact on nurses’ willingness to leave their job.

The results highlight that nurses who regularly face ethically challenging situations, insufficient resources, and high workloads experience increased emotional fatigue and moral strain, which in the long term threatens both their professional stability and patient safety. This situation is not only a matter of staff well-being, but also a systemic challenge to the quality of healthcare.

The results of the study point to the need to introduce workload monitoring instruments (e.g., NAS) and to develop structured support mechanisms for nurses, especially in ICU settings. It is also important to review nurse-to-patient ratios at the national level to reduce chronic overload. At the institutional level, the professional environment needs to be strengthened through emotional and ethical support, supervision, and participation in decision-making.

This study serves as a basis for further, in-depth research and to inform policy makers on how working conditions affect staff retention and the quality of patient care. Targeted action on these issues can contribute to a sustainable healthcare system where intensive care nurses feel protected, motivated, and professionally supported.

## Figures and Tables

**Table 1:** Mean NAS and staff shortage by hospital, level, and shift.

Hospital	Shift	Mean	SD	Min	Max	Current Number of Nurses	Nurse Shortage (M)	Nurse Shortage (%)
A	D	58.38	20.14	21.40	136.50	8	0.97	32.4
	N	58.13	19.20			8	0.95	31.7
B	D	106.96	18.83	33.30	156.90	2	2.46	82.1
	N	105.18	18.99			2	2.19	73.0
C	D	71.31	24.61	28.80	154.30	2	1.32	44.0
	N	70.85	19.69			2	1.14	38.0

**Table 2:** Aggregated Unit-Level Indicators (By Hospital and Shift) *.

Hospital	Shift	Mean	SD	Nurse Shortage (M)	MMD-HP (Mean) *	CBI (Mean) *	ATS (Mean) *
A	D	58.38	20.14	32.4	47.6	45.4	38.9
	N	58.13	19.20	31.7			
B	D	106.96	18.83	82.1	54.7	53.8	45.1
	N	105.18	18.99	73.0			
C	D	71.31	24.61	44.0	50.1	48.9	42.1
	N	70.85	19.69	38.0			

* Note: The indicators on moral distress, burnout, and staff turnover were obtained in a one-off survey and refer to each unit as a whole, not to specific shifts.

## Data Availability

The datasets produced and examined in this study can be obtained from the corresponding author upon a reasonable request. All data generated or analyzed during this study are provided, within the published article. The data utilized in this study are confidential.

## Abbreviations

The following abbreviations are used in this manuscript:

EU	European Union
ICU	Intensive Care Units
NAS	The Nursing Activities Score
r	Pearson Correlation Coefficient
COVID-19	Coronavirus Disease 2019
USA	United States of America
TISS-28	Therapeutic Intervention Scoring System—28 items
MMD-HP	Moral Distress Scale for Healthcare Professionals
CBI	Copenhagen Burnout Inventory
ATS	Anticipated Turnover Scale
IBM SPSS	Statistical Package for the Social Sciences
ANOVA	Analysis of Variance
PĒK	Pētījuma Ētikas komitēja
D	Day
N	Night
SD	Standard Deviation
min	Minimum
max	Maximum
M	Mean
PRB	Personal-related burnout
WRB	Work-related burnout
CRB	Client-related burnout
SE	Standard Error

## References

[1] European Commission (2021). State of Health in the EU: Latvia—Country Health Profile 2021.

[2] OECD (2023). Nurses (Indicator). OECD Data. https://data.oecd.org/healthres/nurses.htm.

[3] National Health Service of Latvia (NVD) (2024). Statistikas Pārskats Par Ārstniecības Personām un Ārstniecības Atbalsta Personām uz 2023. Gada 31. Decembri. Nacionālais Veselības Dienests, Rīga. https://www.vmnvd.gov.lv/sites/vmnvd/files/data_content/58b57889c736b1.pdf.

[4] Arteaga G., Bacu L., Moreno Franco P. (2023). Patient Safety in the Critical Care Setting: Common Risks and Review of Evidence-Based Mitigation Strategies. Contemporary Topics in Patient Safety—Volume 2.

[5] Ervin J.N., Kahn J.M., Cohen T.R., Weingart L.R. (2018). Teamwork in the Intensive Care Unit. Am. Psychol..

[6] Cerela-Boltunova O., Millere I., Trups-Kalne I. (2024). Adaptation of the Nursing Activities Score in Latvia. Int. J. Environ. Res. Public Health.

[7] Maghsoud F., Rezaei M., Asgarian F.S., Rassouli M. (2022). Workload and Quality of Nursing Care: The Mediating Role of Implicit Rationing of Nursing Care, Job Satisfaction and Emotional Exhaustion by Using Structural Equations Modeling Approach. BMC Nurs..

[8] Edú-Valsania S., Laguía A., Moriano J.A. (2022). Burnout: A Review of Theory and Measurement. Int. J. Environ. Res. Public Health.

[9] Lewis S., Willis K., Smallwood N. (2025). The Collective Experience of Moral Distress: A Qualitative Analysis of Perspectives of Frontline Health Workers during COVID-19. Philos. Ethics Humanit. Med..

[10] Hwang Y.-S., Kim B.-J. (2021). “The Power of a Firm’s Benevolent Act”: The Influence of Work Overload on Turnover Intention, the Mediating Role of Meaningfulness of Work and the Moderating Effect of CSR Activities. Int. J. Environ. Res. Public Health.

[11] Alghamdi M.G. (2016). Nursing Workload: A Concept Analysis. J. Nurs. Manag..

[12] Juvé-Udina M.E., González-Samartino M., López-Jiménez M.M., Planas-Canals M., Rodríguez-Fernández H., Batuecas Duelt I.J., Tapia-Pérez M., Pons Prats M., Jiménez-Martínez E., Barberà Llorca M.À. (2020). Acuity, nurse staffing and workforce, missed care and patient outcomes: A cluster-unit-level descriptive comparison. J. Nurs. Manag..

[13] Kalisch B.J., Landstrom G.L., Hinshaw A.S. (2009). Missed nursing care: A concept analysis. J. Adv. Nurs..

[14] Lief L., Berlin D.A., Maciejewski R.C., Westman L., Su A., Cooper Z.R., Ouyang D.J., Epping G., Derry H., Russell D. (2018). Dying Patient and Family Contributions to Nurse Distress in the ICU. Ann. Am. Thorac. Soc..

[15] Goudarzian A.H., Nikbakht Nasrabadi A., Sharif-Nia H., Farhadi B., Navab E. (2024). Exploring the Concept and Management Strategies of Caring Stress among Clinical Nurses: A Scoping Review. Front. Psychiatry.

[16] Morley G., Ives J., Bradbury-Jones C., Irvine F. (2019). What Is ‘Moral Distress’? A Narrative Synthesis of the Literature. Nurs. Ethics.

[17] Orgambídez A., Borrego Y., Alcalde F.J., Durán A. (2025). Moral Distress and Emotional Exhaustion in Healthcare Professionals: A Systematic Review and Meta-Analysis. Healthcare.

[18] Burrowes S.A.B., Casey S.M., Pierre-Joseph N., Talbot S.G., Hall T., Christian-Brathwaite N., Del-Carmen M., Garofalo C., Lundberg B., Mehta P.K. (2023). COVID-19 Pandemic Impacts on Mental Health, Burnout, and Longevity in the Workplace among Healthcare Workers: A Mixed Methods Study. J. Interprof. Educ. Pract..

[19] Calkins K., Guttormson J., McAndrew N.S., Losurdo H., Loonsfoot D., Schmitz S., Fitzgerald J. (2023). The Early Impact of COVID-19 on Intensive Care Nurses’ Personal and Professional Well-Being: A Qualitative Study. Intensive Crit. Care Nurs..

[20] Ge M.-W., Hu F.-H., Jia Y.-J., Tang W., Zhang W.-Q., Zhao D.-Y., Shen W.-Q., Chen H.-L. (2023). COVID-19 Pandemic Increases the Occurrence of Nursing Burnout Syndrome: An Interrupted Time-Series Analysis of Preliminary Data from 38 Countries. Nurse Educ. Pract..

[21] Vogt K.S., Simms-Ellis R., Grange A., Griffiths M.E., Coleman R., Harrison R., Shearman N., Horsfield C., Budworth L., Marran J. (2023). Critical Care Nursing Workforce in Crisis: A Discussion Paper Examining Contributing Factors, the Impact of the COVID-19 Pandemic and Potential Solutions. J. Clin. Nurs..

[22] Gibney R.T.N., Blackman C., Gauthier M., Fan E., Fowler R., Johnston C., Jeremy Katulka R., Marcushamer S., Menon K., Miller T. (2022). COVID-19 Pandemic: The Impact on Canada’s Intensive Care Units. FACETS.

[23] Dalmolin G.D.L., Lunardi V.L., Lunardi G.L., Barlem E.L.D., Silveira R.S.D. (2014). Moral Distress and Burnout Syndrome: Are There Relationships between These Phenomena in Nursing Workers?. Rev. Lat.-Am. Enferm..

[24] Gupta J. (2024). Employee Well-Being Initiatives: A Critical Analysis Of HRM Practices. Educ. Adm. Theory Pract..

[25] Li L., Zou X., Chen H. (2025). Workload in ICU Nurses: A Systematic Review and Meta-Analysis of the Nursing Activities Score. Intensive Crit. Care Nurs..

[26] Miranda D.R., Nap R., de Rijk A., Schaufeli W., Iapichino G., Members of the TISS Working Group (2003). Nursing Activities Score. Crit. Care Med..

[27] Cerela-Boltunova O., Paskova A. (2025). Moral distress among neonatal and pediatric intensive care nurses before and during COVID-19: A systematic review. PREPRINT (Version 1) available at Research Square. Res. Sq..

[28] Tamata A.T., Mohammadnezhad M. (2023). A Systematic Review Study on the Factors Affecting Shortage of Nursing Workforce in the Hospitals. Nurs. Open.

[29] Cerela-Boltunova O., Millere I., Nagle E. (2025). Moral Distress, Professional Burnout, and Potential Staff Turnover in Intensive Care Nursing Practice in Latvia—Phase 1. Int. J. Environ. Res. Public Health.

[30] Cerela-Boltunova O., Paskova A. (2024). Adaptation and Validation of the Moral Distress Scale-Healthcare Professionals in Latvia. J. Ecohumanism.

[31] Cerela-Boltunova O., Millere I., Trups I. (2025). Adaptation of the Copenhagen Burnout Inventory in Latvia: Psychometric Data and Factor Analysis. Int. J. Environ. Res. Public Health.

[32] Gerber R.M., Hinshaw A.S., Atwood J.R. (1983). Anticipated turnover among nursing staff. Ariz. Nurse.

[33] Raosoft, Inc Sample Size Calculator. http://www.raosoft.com/samplesize.html.

[34] Hayes A.F. (2022). Introduction to Mediation, Moderation, and Conditional Process Analysis: A Regression-Based Approach.

[35] World Medical Association (2013). World Medical Association Declaration of Helsinki: Ethical Principles for Medical Research Involving Human Subjects. JAMA.

[36] Ramírez-Elvira S., Romero-Béjar J.L., Suleiman-Martos N., Gómez-Urquiza J.L., Monsalve-Reyes C., Cañadas-De La Fuente G.A., Albendín-García L. (2021). Prevalence, Risk Factors and Burnout Levels in Intensive Care Unit Nurses: A Systematic Review and Meta-Analysis. Int. J. Environ. Res. Public Health.

[37] Banda Z., Simbota M., Mula C. (2022). Nurses’ Perceptions on the Effects of High Nursing Workload on Patient Care in an Intensive Care Unit of a Referral Hospital in Malawi: A Qualitative Study. BMC Nurs..

[38] Dewi S.P., Susanti M. (2021). Effect of Work Overload on Job Satisfaction Through Burnout. J. Manaj..

[39] Rodriguez-Ruiz E., Campelo-Izquierdo M., Veiras P.B., Rodríguez M.M., Estany-Gestal A., Hortas A.B., Rodríguez-Calvo M.S., Rodríguez-Núñez A. (2022). Moral Distress among Healthcare Professionals Working in Intensive Care Units in Spain. Med. Intensiv. (Engl. Ed.).

[40] Llaurado-Serra M., Santos E.C., Grogues M.P., Constantinescu-Dobra A., Coţiu M.-A., Dobrowolska B., Friganović A., Gutysz-Wojnicka A., Hadjibalassi M., Ozga D. (2025). Critical Care Nurses’ Intention to Leave and Related Factors: Survey Results from 5 European Countries. Intensive Crit. Care Nurs..

[41] Villagran C.A., Dalmolin G.D.L., Barlem E.L.D., Greco P.B.T., Lanes T.C., Andolhe R. (2023). Association between Moral Distress and Burnout Syndrome in University-Hospital Nurses. Rev. Lat.-Am. Enferm..

[42] Salari N., Shohaimi S., Khaledi-Paveh B., Kazeminia M., Bazrafshan M.-R., Mohammadi M. (2022). The Severity of Moral Distress in Nurses: A Systematic Review and Meta-Analysis. Philos. Ethics Humanit. Med..

[43] Li L.Z., Yang P., Singer S.J., Pfeffer J., Mathur M.B., Shanafelt T. (2024). Nurse Burnout and Patient Safety, Satisfaction, and Quality of Care: A Systematic Review and Meta-Analysis. JAMA Netw. Open.

[44] Bruyneel A., Smith P., Tack J., Pirson M. (2021). Prevalence of Burnout Risk and Factors Associated with Burnout Risk among ICU Nurses during the COVID-19 Outbreak in French Speaking Belgium. Intensive Crit. Care Nurs..

[45] Xu G., Zeng X., Wu X. (2023). Global Prevalence of Turnover Intention among Intensive Care Nurses: A Meta-analysis. Nurs. Crit. Care.

[46] Lee J.J., Ji H., Lee S., Lee S.E., Squires A. (2024). Moral Distress, Burnout, Turnover Intention, and Coping Strategies among Korean Nurses during the Late Stage of the COVID-19 Pandemic: A Mixed-Method Study. J. Nurs. Manag..

[47] Bolado G.N., Ataro B.A., Gadabo C.K., Ayana A.S., Kebamo T.E., Minuta W.M. (2024). Stress Level and Associated Factors among Nurses Working in the Critical Care Unit and Emergency Rooms at Comprehensive Specialized Hospitals in Southern Ethiopia, 2023: Explanatory Sequential Mixed-Method Study. BMC Nurs..

[48] Bruyneel A., Tack J., Droguet M., Maes J., Wittebole X., Miranda D.R., Pierdomenico L.D. (2019). Measuring the Nursing Workload in Intensive Care with the Nursing Activities Score (NAS): A Prospective Study in 16 Hospitals in Belgium. J. Crit. Care.

[49] Kelly L.A., Gee P.M., Butler R.J. (2021). Impact of Nurse Burnout on Organizational and Position Turnover. Nurs. Outlook.

[50] Trusted Health (2024). Front-Line Nurse Career Report: Flexibility Is Key—Findings and Recommendations from Trusted Health’s 2024 Report. TrustedHealth.

[51] National Academies of Sciences, Engineering, and Medicine, National Academy of Medicine (2019). Committee on Systems Approaches to Improve Patient Care by Supporting Clinician Well-Being. Taking Action Against Clinician Burnout: A Systems Approach to Professional Well-Being.

[52] van der Riet P., Levett-Jones T., Aquino-Russell C. (2018). The Effectiveness of Mindfulness Meditation for Nurses and Nursing Students: An Integrated Literature Review. Nurse Educ. Today.

[53] Rushton C.H., Schoonover-Shoffner K., Kennedy M.S. (2017). A Collaborative State of the Science Initiative: Transforming Moral Distress into Moral Resilience in Nursing. Am. J. Nurs..

